# Cervical Multifidus Stiffness Assessment in Individuals with and without Unilateral Chronic Neck Pain: An Inter-Examiner Reliability Study

**DOI:** 10.3390/bioengineering11050500

**Published:** 2024-05-16

**Authors:** Umut Varol, Juan Antonio Valera-Calero, Ricardo Ortega-Santiago, Mónica López-Redondo, Marcos José Navarro-Santana, Gustavo Plaza-Manzano, Pedro Belón-Pérez

**Affiliations:** 1 Escuela Internacional de Doctorado, Universidad Rey Juan Carlos, 28922 Alcorcón, Spain; au.varol.2022@alumnos.urjc.es; 2 Department of Radiology, Rehabilitation and Physiotherapy, Complutense University of Madrid, 28040 Madrid, Spain; juavaler@ucm.es (J.A.V.-C.); marconav@ucm.es (M.J.N.-S.); gusplaza@ucm.es (G.P.-M.); 3 Grupo InPhysio, Instituto de Investigación Sanitaria del Hospital Clínico San Carlos (IdISSC), 28040 Madrid, Spain; 4 Department of Physical Therapy, Occupational Therapy, Rehabilitation and Physical Medicine, Universidad Rey Juan Carlos, 28922 Alcorcón, Spain; ricardo.ortega@urjc.es; 5 Faculty of Health Sciences, Universidad Francisco de Vitoria, 28223 Madrid, Spain; 6 Department of Physical Therapy, Real Madrid C.F., 28055 Madrid, Spain; pebelon@gmail.com

**Keywords:** cervical multifidus muscle, chronic neck pain, diagnostic accuracy, shear wave elastography, sonography, ultrasound

## Abstract

This study aimed to evaluate the inter-examiner reliability of shear wave elastography (SWE) for measuring cervical multifidus (CM) muscle stiffness in asymptomatic controls and patients with chronic neck pain. A longitudinal observational study was conducted to assess the diagnostic accuracy of a procedure. SWE images, following a detailed procedure previously tested, were acquired by two examiners (one novice and one experienced) to calculate the shear wave speed (SWS) and Young’s modulus. The painful side was examined for the experimental cases while the side examined in the control group was selected randomly. Data analyses calculated the intra-class correlation coefficients (ICCs), absolute errors between examiners, standard errors of measurement, and minimal detectable changes. A total of 125 participants were analyzed (*n* = 54 controls and *n* = 71 cases). The Young’s modulus and SWS measurements obtained by both examiners were comparable within the asymptomatic group (both, *p* > 0.05) and the chronic neck pain group (both, *p* > 0.05). Nonetheless, a notable distinction was observed in the absolute error between examiners for shear wave speed measurements among patients with neck pain, where a significant difference was registered (*p* = 0.045), pointing to a sensitivity in measurement consistency affected by the presence of chronic neck pain. ICCs demonstrated moderate-to-good reliability across both groups, with ICC values for asymptomatic individuals reported as >0.8. Among the chronic neck pain patients, ICC values were slightly lower (>0.780). The study revealed moderate-to-good consistency, highlighting the practicality and generalizability of SWE.

## 1. Introduction

The cervical multifidus plays a key role in the stabilization of the cervical spine. It originates primarily from the superior articular processes of C4 to C7 vertebrae and attaches to the spinous processes two to four segments above the origin [1]. It is anatomically located deep to the semispinalis cervicis muscle, lateral to the junction of the spinous process and the lamina, dorsal to the lamina of the vertebrae, and medial to the articular pillar [1]. The muscle’s structure is characterized by various lengths of muscle–tendon units and fascicles, contributing to its functional capabilities for force generation in extension, lateral bending, and axial rotation movements [2]. 

Several studies confirmed its clinical relevance in patients with neck pain as findings included muscle size (decreased cross-sectional area specifically at the affected side and level) and histology (increased intramuscular fatty infiltrates which correlates with pain-related disability) [3,4,5,6]. However, some of these findings are controversial and might depend on the neck pain etiology, duration, and severity [7]. Additionally, the measurement of cervical multifidus contraction patterns using ultrasound imaging (US) has been explored, describing how healthy subjects exhibited a consistent and predictable pattern of cervical multifidus muscle thickness increase during isometric head extension exercises and how, in contrast, patients with chronic neck pain showed altered, less predictable cervical multifidus contraction patterns, suggesting potential dysfunction or weakness in the muscle [8]. 

In addition to brightness mode (B-mode) US, which is used for assessing the cervical multifidus morphological and histological characteristics [9], recent advances in shear wave elastography provided new insights into the evaluation of muscle stiffness [10], offering valuable data in various clinical contexts. A previous study focused on assessing muscle stiffness in the context of idiopathic chronic neck pain [11] concluded that this metric is crucial as it may reflect changes in neuromuscular control and muscle material properties since their results revealed that individuals with chronic neck pain exhibited altered stiffness in specific muscles compared to healthy controls. This difference in muscle stiffness, independent of muscle activity, emphasizes the importance of evaluating both stiffness and activation in diagnosing and designing rehabilitation programs for neck pain sufferers and may lead to more tailored and effective treatment strategies. Although its application in evaluating the lumbar multifidus muscle in patients with unilateral lumbar disk herniation has shown promising results [12], the cervical region remains less explored.

Hence, this study aimed to analyze the inter-examiner reliability of shear wave elastography for assessing the cervical multifidus stiffness using a reproducible US protocol previously described [13] in healthy individuals and patients with mechanical chronic neck pain. 

## 2. Materials and Methods

### 2.1. Study Design

From November 2022 to April 2023, a diagnostic accuracy study with a cross-sectional observational design was carried out at a private university. This research was conducted in accordance with the Guidelines for Reporting Reliability and Agreement Studies (GRRAS) [14] and the Enhancing the QUAlity and Transparency Of health Research (EQUATOR) guidelines [15] to ensure high standards of scientific writing and methodology. Prior to the commencement of data collection, the study’s protocol and ethical considerations were reviewed, approved, and monitored by an Ethics Committee (URJC 1602202307923).

### 2.2. Participants

Local notifications were distributed throughout the Faculty of Health Sciences to attract two distinct groups: one of asymptomatic individuals and another of individuals suffering from unilateral chronic idiopathic neck pain. The primary requirement for participation was being between the ages of 18 and 65. Exclusion criteria included individuals experiencing neck pain due to trauma, those on muscle relaxants or other drugs affecting muscle tone, and individuals with a history of neck surgery, any neuropathic disorders, or significant degenerative changes observed in radiological exams [16]. 

To qualify for the asymptomatic group, candidates had to confirm no neck pain in the past year. For the chronic pain group, participants had to report unilateral mechanical neck pain (a subset of neck pain labeled as “neck pain with mobility deficits” described in the classification proposed by a recognized Clinical Practice Guideline for the diagnosis and management of neck pain [17]), with at least one clinically relevant episode during the previous year and a minimum average pain intensity of 4 on the Visual Analogue Scale (where scores above 3.5 out of 10 are deemed moderate) [18]. Patients fitting in any other subset (e.g., “neck pain with movement coordination impairments (whiplash associated disorders)”, “neck pain with headache (cervicogenic)”, or “neck pain with radiating pain (radicular)”) [17] were excluded from the study.

All potential participants had to read and sign an informed consent document before they could be included in the study’s data gathering phase.

### 2.3. Sample Size Estimation

The minimal sample size for this research was calculated following Walter et al.’s guidelines [16], which utilize intraclass correlation coefficients (ICCs). This calculation was based on two reliability studies [17,18] that employed US and shear wave elastography on the cervical multifidus muscle in both neck pain sufferers and non-sufferers, with ICCs ranging between 0.63 and 0.99 as the reference score. Given that inter-examiner reliability is usually less consistent than intra-examiner reliability for assessing this structure [12,19], an ICC greater than 0.70—recognized as indicative of good reliability [20]—was set as the minimum threshold.

Considering (1) an anticipated ICC value of 0.9, derived from B-mode US reliability findings; (2) a statistical power of 80% and a 5% significance threshold; and (3) an estimated 10% attrition rate due to the longitudinal design of the study (participants were evaluated twice, with a significant interval between sessions), the required sample size was established at a minimum of 65 data points.

### 2.4. Patient-Reported Outcome Measures

Two instruments were used for assessing pain intensity and pain-related disability in the group of cases with neck pain:

The Visual Analogue Scale was used for assessing pain intensity. This method consists of a 10 cm horizontal line where the left end of the line represents “no pain” and the right end represents “worst imaginable pain”. Patients are asked to mark the level of pain on the line, which provides a quantitative measure of pain intensity from 0 to 10 cm. A previous review highlighted its good-to-excellent test–retest reliability supported by high-to-moderate-quality evidence when used with patients experiencing neck pain [19]. A mean average of 3 measures (current pain intensity and the worst and lowest pain intensity during the previous week) was calculated for the analyses.

Since the Visual Analogue Scale showed limited validity to estimate pain-related disability in patients suffering chronic musculoskeletal pain [20], the Neck Disability Index was used for this purpose as a previous meta-analysis reported good internal consistency and test–retest reliability without a floor or ceiling effect [21]. This self-reported questionnaire consists of 10 items that confer various aspects of daily life activities affected by neck pain in a 6-point Likert scale (where 0 indicates no disability and 5 indicates complete disability related to that specific activity). Final scores range from 0 to 100, where higher scores represent greater disability.

### 2.5. Examiners

This study involved two examiners: one with over a decade of experience in musculoskeletal US and clinical expertise in musculoskeletal conditions and chronic neck pain, and another with one year of experience in US. This arrangement aimed to evaluate how examiner expertise affects measurement concordance, reflecting typical clinical settings where professionals of varied experience levels work together. To ensure methodological rigor, participant involvement and the examination side (left or right) were randomized. A schedule with alternating shifts (9:00 a.m. to 1:00 p.m. and 3:00 p.m. to 5:00 p.m.) was set up to prevent interaction and maintain consistent decision-making, with shift rotations occurring daily. Participants were required to have two appointments on the same day, one in each shift, to be examined by both professionals.

Each examiner encoded the images they acquired, following guidelines from the lead researcher. Both the experienced and novice examiners then independently measured their own images, randomizing the sequence (participant and side). This approach was chosen to yield more practical, clinically relevant results, as it is common in clinical settings for the same examiner to analyze their captured images. The blinding process took into account three factors for each image: the examiner’s experience level, the participant’s status (case or control), and the examination side.

### 2.6. Images Acquisition

Ultrasound images were captured using a Logiq E9 ultrasound system equipped with a linear transducer (6–15 MHz ML-6-15-D, General Electric Healthcare, Milwaukee, WI, USA). For each imaging session, the device was set to standard console parameters, specifically a frequency of 12 MHz, a gain setting of 65 dB, a scanning depth set at 4.5 cm, and the focal zone positioned at the level of the cervical multifidus. For the group experiencing discomfort, ultrasound images were obtained from the painful side, whereas, for the asymptomatic group, the side to be scanned was chosen at random.

During the ultrasound examinations, participants lay in the prone position with a pillow strategically positioned under their ankles to ensure a passive and neutral alignment of the cranio-cervical spine. Their arms were positioned at 90 degrees of abduction and their elbows were bent at 90 degrees. To minimize variability caused by muscle tension, all individuals were instructed to keep their muscles relaxed throughout the imaging process.

**Figure 1:**
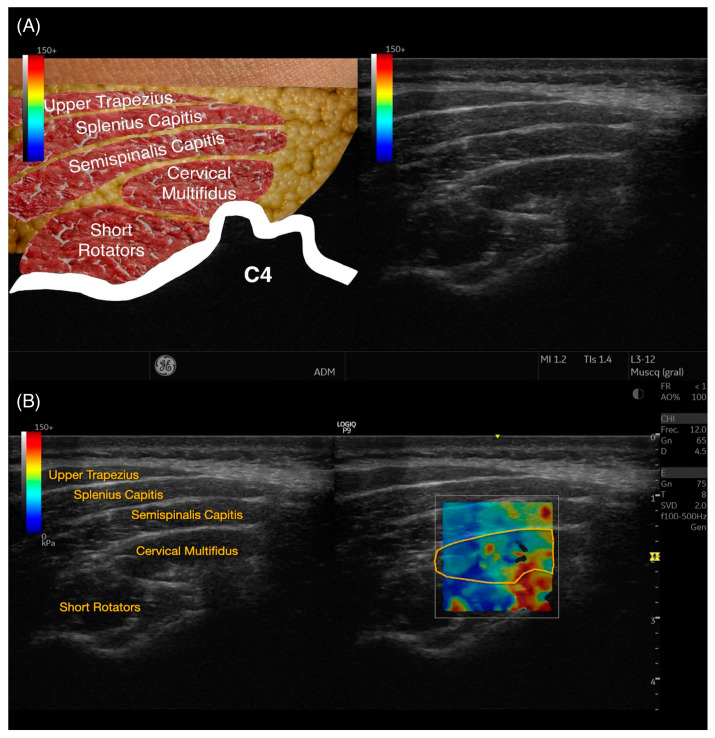
Raw ultrasound imaging acquired at C4–C5 level (**A**), contouring the targeted structure (cervical multifidus) and references used (articular pillar of C4–C5), and shear wave elastography imaging (**B**).

The approach for locating the cervical multifidus muscle adhered to the protocol established by Valera-Calero et al. [13] targeting the C4–C5 vertebral level. This specific protocol was selected due to its precise guidance on locating the region of interest, outlining the contour, and its proven high reliability in previous studies. The process began with manual palpation of the C2 spinous process. The transducer was then aligned horizontally to obtain a B-mode short-axis view starting at C2. A downward (caudal) glide of the transducer continued until the C4 vertebra was visualized. Subsequently, a lateral glide was executed to focus on the C4 over the articular pillar. The imaging captured the cervical multifidus muscle at the point where the most superficial aspect of the spinous tubercle’s cortical surface and the topmost point of the C4/C5 joint were simultaneously visible (Figure 1). Particular attention was paid to ensure that the muscle was visualized perpendicularly at the center of the image while applying the least amount of pressure necessary to obtain a clear view.

Subsequently, all collected images were processed using the offline software of the Logiq E9 ultrasound system. This analysis involved the meticulous outlining of the cervical multifidus muscle’s perimeter, taking care to exclude any bone, nerve roots, or adjacent fascia, as demonstrated in Figure 1. Following the contouring, calculations for Young’s modulus and the shear wave speed (SWS) were automatically generated by the software, providing essential biomechanical properties of the muscle.

### 2.7. Statistical Analysis

Data processing and analysis utilized the Statistical Package for the Social Sciences (SPSS), version 27 for Mac OS (Armonk, NY, USA). The significance threshold for all statistical tests was set at a two-tailed *p*-value of less than 0.05. To determine the normality of the distribution of continuous variables, histograms and Shapiro–Wilk tests were employed.

Descriptive statistics were then applied to detail the sociodemographic attributes and US characteristics of the participants. Differences between genders for demographic and US variables, as well as side differences for US data, were analyzed using Student’s *t*-test, which included a 95% confidence interval.

For assessing the inter-examiner reliability concerning the measurements of Young’s modulus and shear wave speed (SWS), five specific metrics were calculated: (1) the mean average and standard deviation obtained from both examiners; (2) the absolute error between the two examiners; (3) the intraclass correlation coefficients (ICC_3,2_, using a 2-way mixed model for consistency [22]); (4) the standard error of measurement (SEM = standard deviation of the mean average × √(1 − ICC)); and (5) the minimal detectable changes (MDC = SEM × √2 × 1.96) [22].

Finally, regression plots were also created to assess the SWS and Young’s modulus assessment agreement between both examiners [23]. This method provides a visual insight into the consistency and bias between the two measurement methods or observers. For the specific context of this study, the plots were configured with the scores of the novice examiner on the Y-axis and those of the experienced examiner on the X-axis. Each data point in the plot represented a paired measurement of Young’s modulus and shear wave speed (SWS) obtained by the two examiners, in both asymptomatic subjects and individuals with chronic neck pain. The 95% confidence interval was depicted with red lines, providing an estimate of where the true limits of agreement lie, assuming normal distribution of the differences. Meanwhile, the black line represented the regression line, illustrating any systematic bias or trend across the range of measurements. This visual representation helps identify any potential discrepancies and the degree of agreement between the new and experienced examiners [24].

## 3. Results

Out of an initial pool of 130 potential participants, 5 individuals were excluded from the study; 2 (*n* = 2) were excluded because they were asymptomatic but had a prior history of whiplash, while 3 others (*n* = 3) who exhibited symptoms of neck pain were also excluded for the same reason. Consequently, the study proceeded with *n* = 125 participants, of whom 43.2% were asymptomatic and 56.8% were patients actively experiencing symptoms, forming the final sample for data collection.

The demographic description of the sample is available in Table 1. Males with neck pain had comparable BMI and age (both, *p* > 0.05), pain intensity (*p* = 0.229), duration of symptoms (*p* = 0.085), and pain-related disability (*p* = 0.934) compared with females. Between-group differences revealed that cases and controls were comparable in terms of age, body mass, and BMI (all, *p* > 0.05). 

Inter-examiner reliability estimates are summarized in Table 2. The Young’s modulus and SWS obtained by the experienced and the novice examiners did not differ significantly in the asymptomatic cohort (*p* = 0.169 and 0.297, respectively) nor the sample of patients with neck pain (*p* = 0.374 and 0.297, respectively). However, the SWS absolute error between examiners differed significantly in the sample of neck pain patients (*p* = 0.045). Accordingly, ICC scores were moderate-to-good for measuring both metrics for both groups. Data also showed a broad standard deviation, indicating substantial variability. Concerning the precision of shear wave elastography in discerning if score variations in longitudinal research are due to actual changes rather than measurement inaccuracies, the findings indicated higher precision for SWS compared to Young’s modulus.

**Figure 2:**
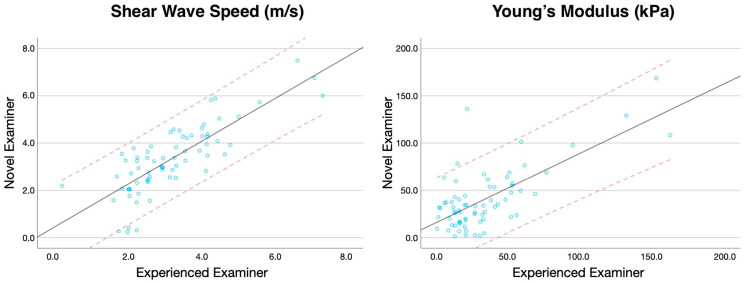
Regression plots comparing the Young’s modulus and shear wave speed score (representing the 95% of Confidence Interval with red dashed lines) obtained by the experienced and novice examiner in asymptomatic subjects.

**Figure 3:**
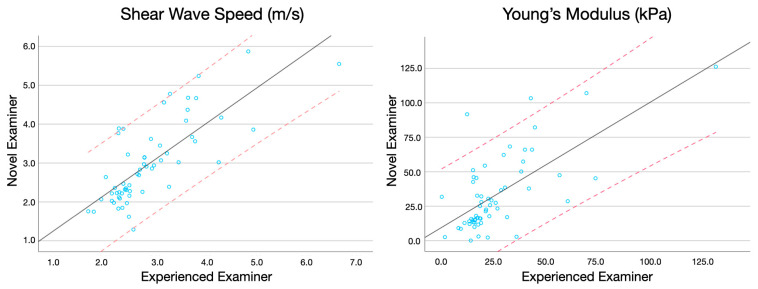
Regression plots comparing the Young’s modulus and shear wave speed score (representing the 95% of Confidence Interval with red dashed lines) obtained by the experienced and novice examiner in patients with chronic neck pain.

Additionally, regression plots illustrating the agreement for measuring Young’s modulus and SWS in healthy individuals and patients with neck pain are available in Figure 2 and Figure 3, respectively.

## 4. Discussion

This investigation represents a pioneering effort in evaluating the reliability of shear wave elastography measurements for assessing muscle stiffness in individuals with and without neck pain. Despite the fact that the stiffness assessment of neck muscles is recommended in patients with neck pain [25,26,27,28,29,30], limited evidence has been utilized to test the reliability of shear wave elastography procedures for measuring the cervical multifidus Young’s modulus and SWS characteristics. After an extensive search, only two studies provided reliability estimates for assessing elasticity properties of deep neck muscles [31,32]. Although their results revealed moderate-to-good reliability in different positions and contraction states, several limitations were disclosed and supported the rationale for conducting this research. 

Young et al. [31] analyzed asymptomatic volunteers. Previous studies assessing the reliability of B-mode US for measuring the cervical multifidus size, shape, and histological characteristics agreed that reliability is poorer in clinical populations compared with asymptomatic controls. The most accepted hypothesis is related to potential difficulties in accurately contouring the muscle as a consequence of smaller muscle and blurred muscle limits attributed to the increased quantity of surrounding connective tissue [33] or inaccurate definition of the cervical multifidus edges. Indeed, the lateral and medial bundle of the semispinalis capitis, semispinalis cervicis, or short rotators are often misdefined as part of the cervical multifidus muscle [1,34,35,36]. 

In addition, only a single examiner was involved in their data collection. To know the inter-examiner reliability of a procedure is crucial as it measures the consistency of test results across different examiners, being especially important in clinical and research settings where assessments are likely to be conducted by various practitioners with different expertise. High inter-examiner reliability ensures that the findings are not dependent on one examiner’s unique approach or interpretation, making the results more generalizable and reliable across different settings, and it helps in establishing the broader applicability of a method or instrument, ensuring that the outcomes are reproducible and valid regardless of who conducts the examination. Finally, the region of interest selected by Young et al. [31] was limited to a circular area of approximately 20 mm^2^ and their scores might not be representative of the entire muscle.

On the other hand, Dieterich et al. [32,37] tested the reliability of shear wave elastography for measuring the posterior cervical muscle stiffness during active tasks and analyzed the differences between cases with neck pain and asymptomatic controls. Regarding the reliability analyses, the results obtained suggested moderate-to-good reliability (ICCs ranged from 0.732 to 0.845, depending on the specific muscle assessed). 

The ICC values obtained in this study were moderate-to-good (above 0.78 for all variables), reflecting a level of consistency that is pivotal for the technique’s clinical application. These findings are consistent with those reported by Amiri-Arimi et al. [6] and Valera-Calero et al. [13,33,38], who also found moderate-to-high reliability in a similar procedure for assessing morphological metrics using B-mode US. The SEM and MDC_95_ values further support the reliability of the measurements, though the relatively high MDC_95_ values, particularly in the group of cases with neck pain, suggest a need for cautious interpretation in longitudinal studies. The high variability observed (as indicated by the standard deviation), especially for the Young’s modulus, across both groups indicates substantial variability in this metric. This variability, as discussed by Yamasaki and Sasaki [39], could be attributed to factors such as muscle heterogeneity and the technical nuances of SWE. Therefore, our recommendation is to use the raw metric (SWS) and avoiding indirect metrics derived from the SWS. 

The good reliability demonstrated in this study has three principal implications. The first one is that our findings adjust the strength of previous research studies’ conclusions evaluating the cervical multifidus stiffness without verifying the procedure reliability. For instance, the shear modulus differences reported by Dieterich et al. [37] between the prone at rest position and head lift condition (14.9 kPa) can be attributed to measurement errors (change was smaller than the MDC cut-off obtained in our reliability analyses) instead of a real change. 

The second implication is the support of using objective and reproducible tools, as Dieterich et al. [32] found a considerably discrepancy between the sensation of stiffness and objective measures of neck muscle stiffness. In their study, the results revealed no significant differences in neck muscle stiffness between women with chronic neck pain and asymptomatic controls.

Finally, shear wave elastography measurements have significant implications, especially when linked to the clinical outcomes of patients with cervical pathologies, such as those discussed in the paper by Sari et al. [40] where active myofascial trigger points were frequently found in patients with cervical radiculopathy. Understanding the frequency and distribution of myofascial trigger points in patients underscores the necessity of accurate and reliable measurement techniques for assessing muscle stiffness and related properties [29,41,42]. In fact, a previous research study described that evaluating the shape patterns of myofascial trigger points (differentiating the nodule itself and surrounding tissues) is a feasible procedure using B-mode US. Future research may analyze the potential stiffness difference between these two locations in active and latent trigger points and determine its association with clinical severity indicators [43]. The high reliability of our shear wave elastography measurements supports the use of these methods for effectively distinguishing between changes in muscle properties due to pathological conditions versus normal variability. Our study’s demonstration of moderate-to-good reliability in shear wave elastography measurements can be directly applied to clinical settings where myofascial trigger points might be evaluated [44,45,46,47,48]. Given that myofascial trigger points are notably prevalent in patients with cervical musculoskeletal conditions [49,50,51,52,53,54], the ability to reliably measure muscle stiffness and elasticity can greatly enhance the diagnostic process. This is crucial not only for the initial assessment but also for monitoring the progression or regression of these points post-treatment [55,56,57]. Furthermore, the association of myofascial trigger points with specific cervical conditions [58] suggests that a targeted approach in treatment could be more effective [59]. Reliable shear wave elastography metrics ensure that treatments aimed at alleviating muscle stiffness are based on accurate assessments, thus potentially improving our understanding in subjacent mechanisms explaining the efficacy of interventions such as dry needling [60,61]. In addition, our results provide a foundation for validating the clinical relevance of changes observed in muscle stiffness, as reported in the existing literature. The ability to discern true pathological changes from those within the bounds of measurement error (as indicated by MDC_95_) is particularly valuable in clinical trials and practice [62]. This capability ensures that clinical decisions and research conclusions are based on robust, reproducible data, thereby enhancing the efficacy of treatments for conditions where muscle stiffness plays a significant role [63].

### Limitations

The main limitation of this study is related to the statistical power of reliability analyses. Despite including an acceptable number of participants, the number of raters was limited, which may affect the generalizability of our findings. Future studies should consider including a larger number of examiners with different levels of expertise to corroborate the findings of this study. Additionally, neck pain etiology plays a relevant role in imaging findings as multiple differences have been reported in the literature between patients with discal herniations, whiplash-associated disorders, or cervical radiculopathies (to list a few examples). Further studies should follow the neck pain classification proposed by clinical practice guidelines [25,26] and analyze separately the results based on this classification.

## 5. Conclusions

This study demonstrates the inter-examiner reliability of shear wave elastography for assessing the stiffness of the cervical multifidus muscle in both asymptomatic individuals and patients with chronic neck pain. The reliability estimates revealed moderate-to-good consistency, highlighting the practicality and generalizability of shear wave elastography in the clinical setting. Despite these results, the study acknowledges certain limitations such as the use of a single US device and a limited number of examiners, suggesting the need for further research with a broader range of equipment and evaluators. The results of this study lay a foundation for the broader application of shear wave elastography in both clinical practice and research, especially in the assessment of muscle stiffness in neck pain patients, thus potentially enhancing diagnostic accuracy and the effectiveness of targeted treatment approaches.

## Figures and Tables

**Table 1:** Participants’ characteristics by group (cases and controls) and gender.

	Sociodemographic Characteristics				Clinical Characteristics		
	Age (y)	Height (m)	Body Mass (kg)	BMI (kg/m^2^)	VAS (0–10)	Pain Duration (Months)	NDI (0–100)
Asymptomatic subjects (*n* = 54)	20.8 ± 2.7	1.70 ± 0.09	69.4 ± 18.4	23.9 ± 5.7			
Males (*n* = 40)	21.5 ± 4.8	1.77 ± 0.07 **	74.1 ± 13.5 *	23.5 ± 4.0			
Females (*n* = 14)	19.3 ± 1.6	1.64 ± 0.05	63.7 ± 11.9	23.5 ± 3.6			
Patients with neck pain (*n* = 71)	20.8 ± 4.3	1.73 ± 0.08	71.4 ± 13.8	23.5 ± 3.8	5.9 ± 1.6	9.1 ± 4.4	17.5 ± 9.3
Males (*n* = 25)	21.5 ± 3.0	1.80 ± 0.05 **	82.5 ± 22.3 **	25.5 ± 7.6	5.56 ± 1.53	10.5 ± 5.2	17.4 ± 10.2
Females (*n* = 46)	20.5 ± 2.5	1.64 ± 0.06	62.3 ± 10.6	23.1 ± 4.1	6.04 ± 1.69	8.4 ± 3.9	17.6 ± 8.7
Case–Control Difference	0.0 (−1.3; 1.2) *p* = 0.927	0.03 (0.00; 0.07) *p* = 0.021	2.0 (−3.9; 7.9) *p* = 0.504	0.4 (−1.4; 2.2) *p* = 0.648			

Significant differences between genders: * *p* < 0.05; ** *p* < 0.001.

**Table 2:** Inter-examiner reliability for the stiffness assessment of the cervical multifidus muscle in asymptomatic and neck pain individuals.

Variables	Asymptomatic Individuals (*n* = 54)		Patients with Neck Pain (*n* = 71)	
	Young’s Modulus (kPa)	Shear Wave Speed (m/s)	Young’s Modulus (kPa)	Shear Wave Speed (m/s)
Mean	30.0 ± 21.9	2.94 ± 0.90	36.7 ± 29.5	3.27 ± 1.28
Novice Examiner	33.5 ± 27.6	2.99 ± 1.04	40.5 ± 32.8	3.33 ± 1.43
Experienced Examiner	26.5 ± 20.3	2.88 ± 0.87	33.0 ± 31.2	3.21 ± 1.27
Absolute Difference	14.4 ± 16.3	0.48 ± 0.48	17.8 ± 18.6	0.66 ± 0.55
ICC_3,2_ (95% CI)	0.826 (0.719; 0.892)	0.888 (0.821; 0.930)	0.780 (0.630; 0.869)	0.859 (0.762; 0.916)
SEM	9.1	0.30	13.8	0.48
MDC_95_	25.32	0.83	38.35	1.33
CV (%)	48.0	16.3	48.5	20.2

SEM and MDC_95_ are expressed in the units described for each metric.

## Data Availability

The original contributions presented in the study are included in the article, further inquiries can be directed to the corresponding author.
